# Carbon Nanotubes Decrease the Negative Impact of *Alternaria solani* in Tomato Crop

**DOI:** 10.3390/nano11051080

**Published:** 2021-04-22

**Authors:** Yolanda González-García, Gregorio Cadenas-Pliego, Ángel Gabriel Alpuche-Solís, Raúl Iskander Cabrera, Antonio Juárez-Maldonado

**Affiliations:** 1 Doctorado en Ciencias en Agricultura Protegida, Universidad Autónoma Agraria Antonio Narro, Saltillo 25315, Coahuila, Mexico; yolanda_glezg@hotmail.com; 2 Centro de Investigación en Química Aplicada, Saltillo 25294, Coahuila, Mexico; gregorio.cadenas@ciqa.edu.mx; 3 Instituto Potosino de Investigación Científica y Tecnológica, San Luis Potosí 78216, San Luis Potosí, Mexico; alpuche@ipicyt.edu.mx; 4 Department of Plant Biology, Rutgers Agricultural Research and Extension Center (RAREC), Rutgers University, Bridgeton, NJ 08302, USA; cabrera@njaes.rutgers.edu; 5 Departamento de Botánica, Universidad Autónoma Agraria Antonio Narro, Saltillo 25315, Coahuila, Mexico

**Keywords:** carbon nanomaterials, nanotechnology, biotic stress, biostimulation, secondary metabolites, antioxidant compounds, crop growth

## Abstract

The diseases that attack the tomato crop are a limiting factor for its production and are difficult to control or eradicate. Stem and fruit rot and leaf blight caused by *Alternaria solani* causes severe damage and substantial yield losses. Carbon nanotubes (CNTs) could be an alternative for the control of pathogens since they have strong antimicrobial activity, in addition to inducing the activation of the antioxidant defense system in plants. In the present study, multi-walled carbon nanotubes were evaluated on the incidence and severity of *A. solani*. Moreover, to the impact they have on the antioxidant defense system and the photosynthetic capacity of the tomato crop. The results show that the application of CNTs had multiple positive effects on tomato crop. CNTs decreased the incidence and severity of *A. solani*. Furthermore, CNTs increased the fruit yield of tomato crop and dry shoot biomass. The antioxidant system was improved, since the content of ascorbic acid, flavonoids, and the activity of the glutathione peroxidase enzyme were increased. The net photosynthesis and water use efficiency were also increased by the application of CNTs. CNTs can be an option to control *A. solani* in tomato crop, and diminish the negative impact of this pathogen.

## 1. Introduction

Biotic stress caused by pests and diseases puts the productivity of a crop at constant risk since it causes up to 25% of production losses [1]. There are numerous pests and diseases that damage both the quality and quantity of tomato (*Solanum lycopersicum* L.) production. For example, the damage caused by *Alternaria solani* such as stem and fruit rot, and leaf blight, which affect the crop during all stages of the plant’s development [2]. *A. solani* reproduces asexually by multicellular conidia that can form necrotic lesions 2–3 days after infection and reproduce new conidia 3–5 days later, this disease cycle allows a polycyclic infection, and can infect all aerial parts of the plant, including leaves, stem, and fruits. Furthermore, being a necrotrophic fungus, it kills host tissues using the enzymes cellulase and pectin methyl galacturonase and producing numerous toxins [3,4]. The first foliar symptoms in the tomato plant become visible on the lower and older leaves after the emergence of the fungus in the form of dark-colored lesions recognized by their distinctive concentric rings, in addition to necrotic lesions on the stem [5]. On the fruit, it invades the area around the end of the stem causing brown spots with dark concentric circles similar to those of the leaves. Under favorable conditions, mature lesions are usually covered by a black mass of mycelia and fungal spores. Additionally, *A. solani* can cause complete defoliation, substantial yield losses, and plant death if not managed properly [3]. The loss of productivity due to infection by *A. solani* in tomato can be up to 80% [1]. This is due to the fact that it can cause serious damage to the photosynthetic apparatus of the plant by inhibiting the activity of the photosystem II, decreasing the content of chlorophylls, and other photosynthetic pigments, which result in the general inhibition of growth and, therefore, in a severe yield reduction [6].

Although there are numerous chemical pesticides that are commercially available, their prolonged exposure generates multi-resistance in pathogens, in addition to being dangerous for the environment and health. In the current scenario, it has become a challenge to research a chemical pesticide substitute to control multidrug resistant phytopathogens [1].

Carbon nanotubes (CNTs) are laminar structures of hybridized carbon atoms (sp^2^) arranged hexagonally, forming hollow cylindrical tubes, with dimensions of a few nanometers in diameter (up to approximately 100 nm) and lengths in the micrometer range. Depending on the number of layers, they are classified as single-walled CNTs (SWCNT) or multi-walled CNTs (MWCNT) [7]. It is known that the type, size, concentration, and functionalization of CNTs can determine their toxicological and physiological effects in different plant species [8]. CNTs have potential agricultural applications due to their effects on regulating plant growth, the ability to traverse plant cell walls, and as a medium for pesticide application [9]. These can positively influence the metabolism of cells by stimulating enzymatic and metabolic activity and gene expression, in addition to increasing the photosynthetic capacity of the leaves through the increase in the levels of photosynthetic pigments [10]. They are also an alternative for the control of pathogens since they have a great antimicrobial activity, since through direct contact with pathogens, CNTs generate membrane damage and modifications in the cell morphology of the microorganism, and generate oxidative stress induced by reactive oxygen species [11]. In addition, it has been reported that the application of CNTs can lead to an improvement in the productivity of plants both in the hydroponic medium and in soil conditions. However, other studies have shown that CNTs can cause phytotoxic effects such as decreased plant growth, increased generation of reactive oxygen species or decreased weight, or in some cases, CNTs have no effect on different plant species [12].

Derived from the above, the objective of this study was to determine if the application of carbon nanotubes can induce tolerance on *A. solani*, due to its antifungal capacity and the induction of changes in the antioxidant defense system of the plant, and therefore improve the productivity of the tomato crop.

## 2. Materials and Methods

### 2.1. Crop Growth

A greenhouse tomato crop was established using tomato seeds of the “El Cid F1” variety (Harris Moran, Davis, CA, USA), of the saladette type and indeterminate growth. The transplant was carried out in 10 L black polyethylene containers in a mixture of peat moss-perlite substrate in a 1:1 ratio. Steiner solution was used for plant nutrition [13]. The plants were handled on a single stem and developed for 90 days after transplantation (DAT).

### 2.2. Treatments

**Figure 1:**
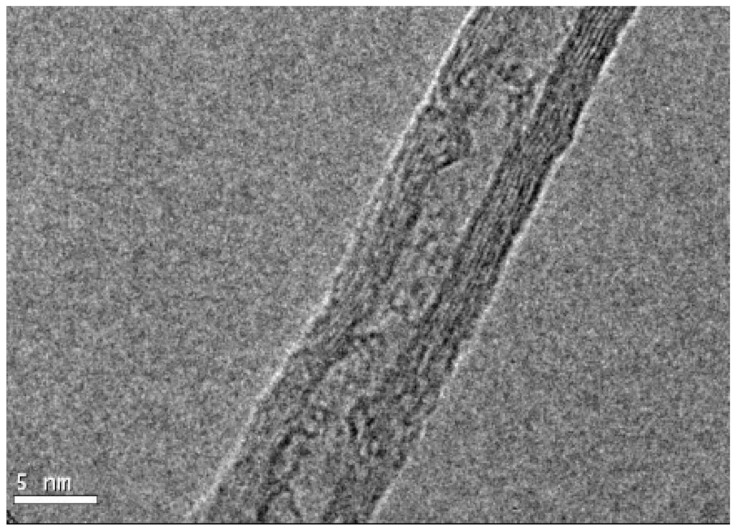
Carbon nanotubes obtained by transmission electron microscopy.

Multi-walled carbon nanotubes (CNTs) with five walls and a diameter of 30–50 nm (Figure 1), with a length of 10–20 µm, and a purity of 95% were used (Nanostructured & Amorphous Materials, Inc., Houston, TX, USA). A dispersion in distilled water was carried out and the Z potential (−39.1 mV) was determined using a Z potential analyzer (ZetaCheck, ZC 0006, Microtrac, Montgomery, PA, USA).

The treatments applied were as follows: (1) Foliar application of carbon nanotubes at a concentration of 100 mg L^−1^ in plants inoculated with *A. solani* (CNTs+Al), (2) copper oxychloride and tetracycline application at a concentration of 1 g L^−1^ in plants inoculated with *A. solani* was used as a commercial control (OX+Al), (3) a positive control inoculated with *A. solani* (Al), (4) carbon nanotubes at a concentration of 100 mg L^−1^ in not inoculated plants (CNTs), and (5) not inoculated plants as a negative control (T0), for a total of five treatments. The CNTs and commercial control were applied by foliar route at intervals of 15 days starting 7 days after transplantation, with a total of five applications. The selection of the concentration and the route of application of the CNTs was based on the results obtained by González-García et al. [14].

### 2.3. Inoculation of A. solani and Analysis of the Incidence and Severity

The *A. solani* spores were reproduced at 29 °C for 15 days in Petri dishes with a Potato Dextrose Agar (PDA) medium supplemented with ampicillin (100 mg mL^−1^). The plants corresponding to the treatments with *A. solani* were inoculated at the time of transplantation with a solution of 1 × 10^7^ spores mL^−1^. For this, 1 mL of solution was injected into the midrib of the first two true leaves, and additionally 4 mL of spore solution were added directly to the substrate for each plant.

The incidence was determined visually considering a positive incidence when the pathogen presented symptoms. The severity of the tomato plants was determined according to the visual scale of Diener and Ausubel [15]. These variables were determined from the inoculation and during the development time of the tomato crop.

### 2.4. Sampling

At 45 days after transplanting, samples were taken from the fully developed third young leaf for biochemical analysis. At 90 DAT, the crop was removed and the fresh shoot biomass was quantified, and dry shoot biomass was obtained after drying for 48 h at 80 °C.

### 2.5. Biochemical Analysis

The chlorophyll content was determined according to the method of Nagata and Yamashita [16], and the data were expressed as milligrams 100 g ^−1^ dry weight (mg 100 g^−1^ DW). The ascorbic acid (mg g^−1^ DW) content was determined by the method described in Padayatty et al. [17]. Glutathione (mmol 100 g^−1^ DW) was determined by the method described in Xue et al. [18], through the reaction of 5,5-ditio-bis-2 nitro benzoic acid (DTNB). Flavonoids (mg 100 g^−1^ DW) were determined by the method described in Arvouet-Grand et al. [19]. Phenols (mg g^−1^ DW) were determined by the method of Folin-Ciocalteu as described in Cumplido-Nájera et al. [20]. The antioxidant capacity was determined with radical DPPH (2,2-difenil-1-picrilhidrazilo) as described in Brand-Williams et al. [21]. For this, the hydrophilic compounds were extracted using a phosphate buffer, and the lipophilic compounds were extracted with a hexane:acetone solution. The total antioxidant capacity was obtained from the sum of the hydrophilic and lipophilic compounds. The antioxidant capacity was expressed as ascorbic acid equivalents (mg g^−1^ DW).

Ascorbate peroxidase (APX)(EC 1.11.1.11) enzyme activity was determined as described in Nakano and Asada [22]. The glutathione peroxidase (GPX)(EC 1.11.1.9) enzyme activity was determined as described in Xue et al. [18]. The catalase (CAT)(EC 1.11.1.6) enzyme activity was determined as described in Dhindsa et al. [23]. In addition, the phenylalanine ammonia lyase (PAL)(EC 4.3.1.5) enzyme activity was determined as described in Sykłowska-Baranek et al. [24]. All of the enzymes were expressed as units per gram of proteins (U g^−1^ Proteins).

### 2.6. Physiological Variables

At 70 days after transplantation, the net photosynthesis rate, intracellular carbon dioxide content, transpiration, and the water use efficiency were determined with a photosynthesis analyzer (3051C, Plant Photosynthesis Meter, Chincan Trading Co. Hangzhou, China).

### 2.7. Statistical Analysis

A Latin square design was used considering five repetitions per treatment, and the analysis of variance and a least significant difference Fisher mean test (α = 0.05) were performed using the InfoStat software (v2018) (Córdoba, Argentina). For the evaluation of incidence and severity, a multivariate analysis of variance (MANOVA) and a Hotelling test (α = 0.05) were performed.

## 3. Results

### 3.1. Incidence and Severity

**Figure 2:**
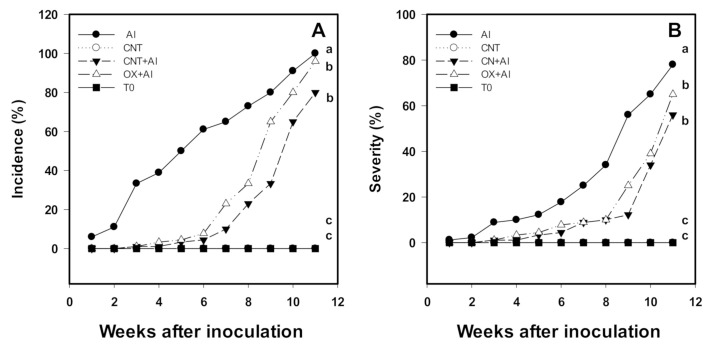
Incidence (**A**) and severity (**B**) of *A. solani* in the tomato crop plants. Al: Positive control inoculated with *A. solani*; CNT: Carbon nanotubes; OX: Commercial control; T0: Control. Different letters indicate significant differences between treatments according to the least significant difference of Fisher test (α = 0.05). N = 5 ± standard error.

The incidence and severity of *A. solani* were modified with the application of CNT (Figure 2). The lowest incidence percentage was observed in the CNT+Al treatment with 80%, the OX+Al treatment had an incidence of 96%, while the positive control showed an incidence of 100%. T0 and CNT had no incidence since they were not inoculated with *A. solani* (Figure 2A).

The severity of *A. solani* decreased with the application of CNTs. With the CNT+Al treatment there was a reduction of up to 44% compared to the positive control. While OX+Al presented a 35% reduction in the severity of the pathogen compared to the positive control (Figure 2B).

### 3.2. Growth of Tomato Plants

**Figure 3:**
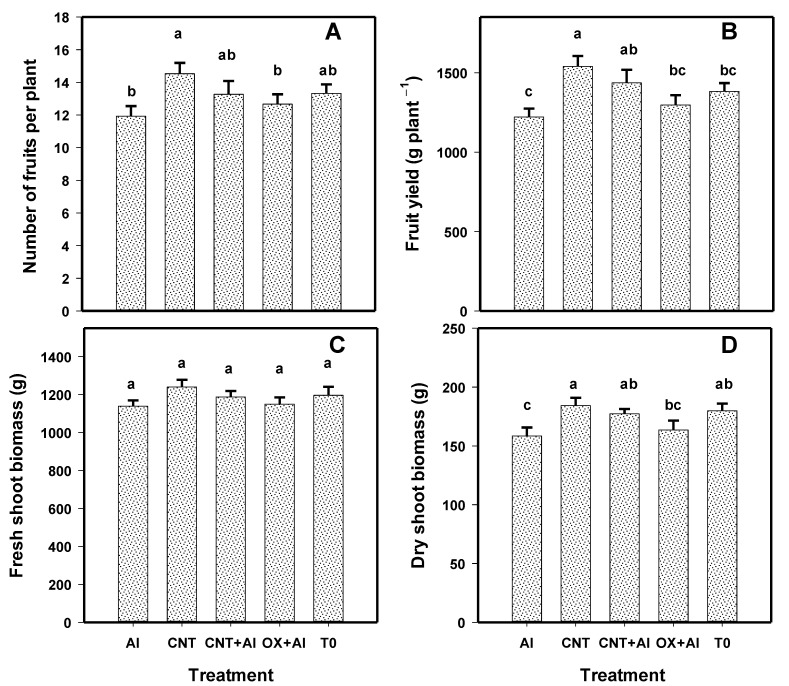
Number of fruits per plant (**A**), fruit yield (**B**), fresh shoot biomass (**C**), and dry shoot biomass (**D**) of tomato crop. Al: Positive control inoculated with *A. solani*; CNT: Carbon nanotubes; OX: Commercial control; T0: Control. Different letters indicate significant differences between treatments according to the least significant difference of Fisher test (α = 0.05). N = 5 ± standard error.

The number of tomato fruits increased with the application of CNTs (Figure 3A). The treatment with the highest number of fruits was CNT, followed by CNT+Al, with 18% and 10%, respectively compared to the positive control. Due to the increase in the number of fruits, the fruit yield in tomato plants also increased. The application of CNT generated a 21% increase in fruit yield, while CNT+Al induced a 15% compared to the positive control (Figure 3B).

The fresh biomass of the plants was not modified with the application of the treatments (Figure 3C). The dry biomass of the plants increased with CNT by 14%, while CNT+Al presented an increase of 10% with respect to the positive control (Figure 3D).

### 3.3. Photosynthetic Pigments

**Figure 4:**
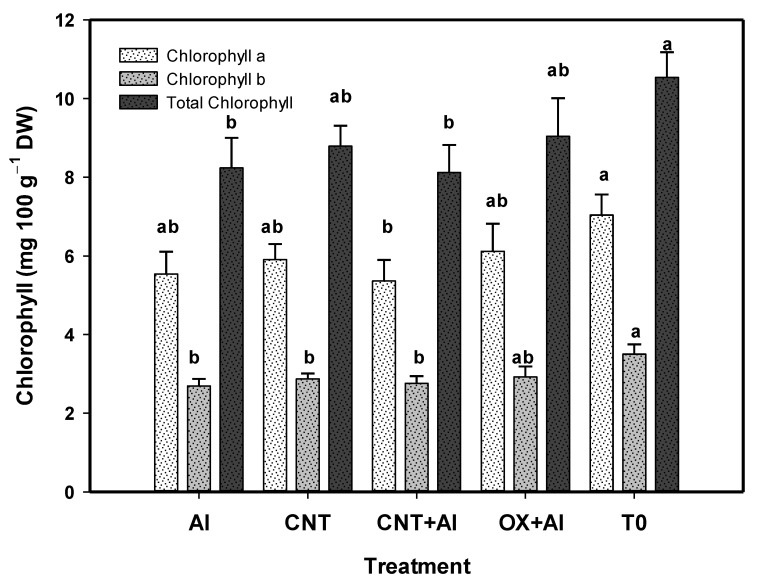
Chlorophylls content in leaves of tomato plants. Al: Positive control inoculated with *A. solani*; CNT: Carbon nanotubes; OX: Commercial control; T0: Control. Different letters indicate significant differences between treatments according to the least significant difference of Fisher test (α = 0.05). N = 5 ± standard error.

The content of photosynthetic pigments showed differences between treatments (Figure 4). The highest content of chlorophyll *a* was observed in T0, while CNT+Al was the one that obtained the lowest content of this compound, however all behaved statistically the same as the positive control. The chlorophyll *b* content was higher in T0, with 23% more than the positive control, the rest of the treatments were statistically equal to the positive control. The total chlorophyll content was higher in T0, with 19% more than the positive control.

### 3.4. Antioxidant Compounds in Leaves

**Figure 5:**
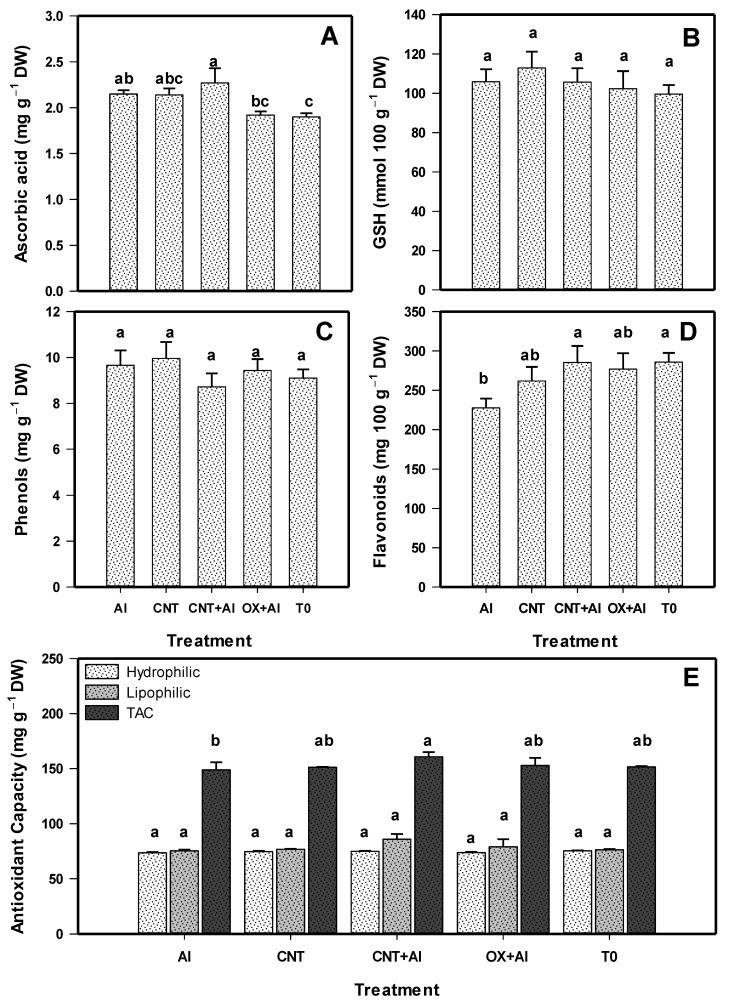
Ascorbic acid (**A**), glutathione (GSH) (**B**), phenols (**C**), flavonoids (**D**), and antioxidant capacity (**E**) in leaves of tomato plants. Al: Positive control inoculated with *A. solani*; CNT: Carbon nanotubes; OX: Commercial control; T0: Control. Different letters indicate significant differences between treatments according to the least significant difference of Fisher test (α = 0.05). N = 5 ± standard error.

The ascorbic acid content was modified with the application of the treatments (Figure 5A). With the CNT+Al treatment, the content of this compound was increased by 5% compared to the positive control, and it also increased by 11% with respect to T0. The glutathione and phenol content did not change with the application of the treatments (Figure 5B,C)

The application of CNTs increased the flavonoid content (Figure 5D). The CNT+Al treatment increased the flavonoid content by 20% compared to the positive control. The antioxidant capacity also showed differences between treatments (Figure 5E). Although no differences were observed between treatments in the antioxidant capacity of hydrophilic and lipophilic compounds, the total antioxidant capacity increased 7% with the CNT+Al treatment compared to the positive control, indicating a positive effect of CNTs in plants inoculated with *A. solani*.

### 3.5. Enzymatic Activity in Leaves

**Figure 6:**
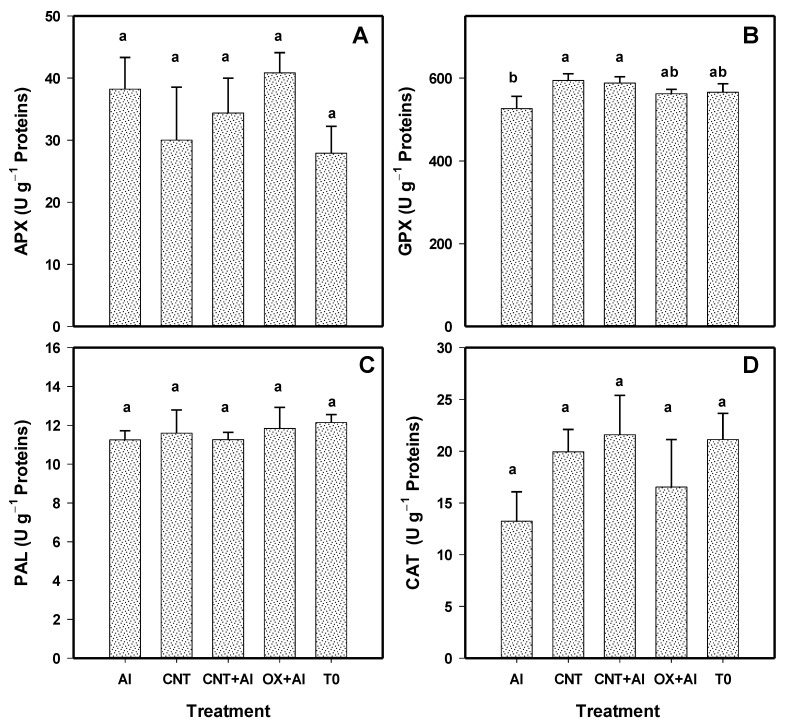
Enzymatic activity of ascorbate peroxidase (APX) (**A**), glutathione peroxidase (GPX) (**B**), phenylalanine ammonia lyase (PAL) (**C**), and catalase (CAT) (**D**) in leaves of tomato plants. Al: Positive control inoculated with *A. solani*; CNT: Carbon nanotubes; OX: Commercial control; T0: Control. Different letters indicate significant differences between treatments according to the least significant difference of Fisher test (α = 0.05). N = 5 ± standard error.

The activity of the APX, PAL, and CAT enzymes was not modified by the application of the treatments (Figure 6A,C,D). Only an 11% increase in GPX activity was observed with CNT and 10% with CNT+Al compared to the positive control (Figure 6B).

### 3.6. Physiological Variables

**Figure 7:**
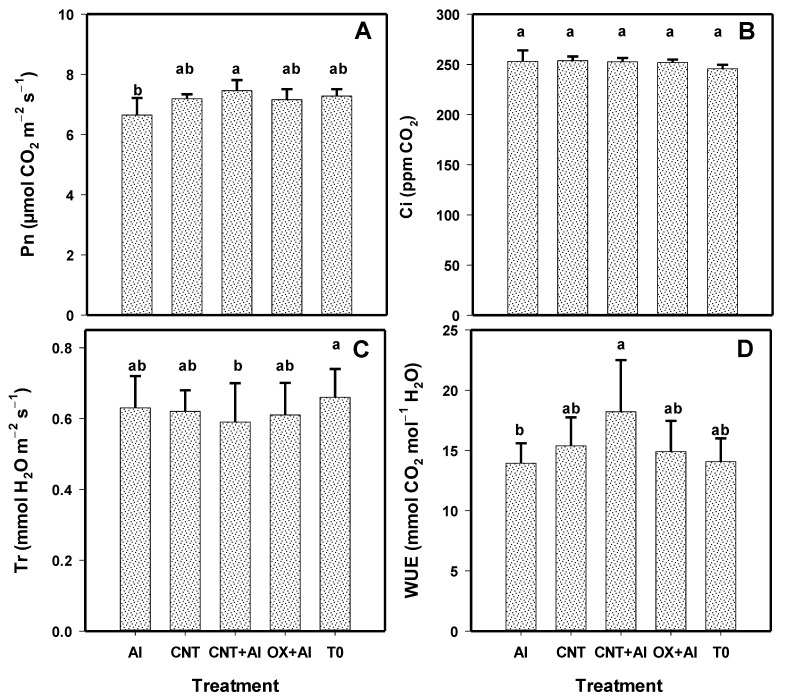
Net photosynthesis rate (Pn) (**A**), internal CO_2_ concentration (Ci) (**B**), transpiration (Tr) (**C**), and water use efficiency (WUE) (**D**) in leaves of tomato plants. Al: Positive control inoculated with *A. solani*; CNT: Carbon nanotubes; OX: Commercial control; T0: Control. Different letters indicate significant differences between treatments according to the least significant difference of Fisher test (α = 0.05). N = 5 ± standard error.

The net photosynthesis rate of tomato plants was modified with the application of the treatments (Figure 7). With the CNT+Al treatment, the net photosynthesis rate of the plants was increased by 11% compared to the positive control (Figure 7A). Stomatal conductance did not change with the application of the treatments (Figure 7B). The highest transpiration rate was observed in T0, while the lowest transpiration rate was observed in the CNT+Al treatment with 8% less than T0 (Figure 7C). Derived from less transpiration, the most efficient treatment in the water use efficiency was CNT+Al, with 23% more than the positive control.

## 4. Discussion

The diseases that attack the tomato crop are a limiting factor for its production and are difficult to control or eradicate [2]. CNTs are an alternative for the control of pathogens in crops, since they present a series of positive effects that range from antifungal capacity through direct effect on microorganisms, as well as the induction of metabolites and defense compounds in plants, and even due to their ability to improve crop growth through increased photosynthesis.

CNTs can enter the plant either by foliar route or through the root. In the foliar route, they can enter through the stomata or through the cuticle, although the entrance is greater by the cuticle due to the large area it has in the leaves compared to the stomata [25]. After their entry, nanomaterials (NMs) reach the tissues of the epidermis and the mesophyll where they can interact with these tissues and their structures, and later they can be translocated to the whole plant through the vascular bundles [26].

When CNTs enter through the root, they can penetrate cell walls, the cytoplasmic membrane, and cross the Caspari strip to enter the vascular xylem and translocate to the rest of the plant [27,28]. In addition, NMs can enter the plant together with water and other solutes, and then move through the transpiration process [29]. In the root, the transport of NMs can be through the apoplast pathway where the transport of larger particles is favored (~200 nm) or through the symplastic pathway where the transport of smaller particles (<50 nm) is favored [30].

Once inside the plant cells, NMs interact with the various cellular organelles which can modify metabolic processes and affect biochemical processes in the cell [29,31]. Furthermore, NMs can move to the nucleus where the interaction can modify the expression of genes related to different physiological processes such as cell division and cell wall elongation [32,33].

CNTs have a strong antimicrobial activity that implies physical and chemical damage. Physical damage is induced through direct contact causing damage to the membrane and modifications in cell morphology generating leakage of cytoplasmic material, release of enzymes, and electrolytes and degradation of lipids that result in the cell death of the microorganism [11,34]. In addition, the size of the CNTs is of great relevance in the deactivation of microorganisms, since being small increases their surface/volume ratio, which results in a stronger bond with the cell wall or membrane of microorganisms, exerting their action more effectively [35]. The so-called “capture of microorganisms” is another antibacterial mechanism of carbon-based materials, since their lamellar structures can interact with bacteria and easily cover their surfaces, block active sites, and decrease the viability of microorganisms, since they are trapped and inactive since they remain isolated from their environment and cannot proliferate [36].

One of the main chemical effects caused by CNTs is the oxidative stress generated by reactive oxygen species, since the interactions of nanomaterials with microorganisms lead to the transfer of electrons generating oxidative stress and consequently biological death [37]. Siddiqui et al. [38] demonstrated under in vitro conditions that the addition to the culture medium of 0.05 and 0.10 mg mL^−1^ of graphene oxide inhibited the growth of the pathogens *Pectobacterium carotovorum*, *Xanthomonas campestris* pv. *Carotae*, *Alternaria dauci*, and *Fusarium solani*, in addition to reducing hatching and increasing mortality of the root-knot nematode *Meloidogyne javanica*. Wang et al. [39] reported the antifungal activity of CNTs against the plant pathogens *F. graminearum* and *F. poae* when adding 62.5–500 μg mL^−1^ in potato dextrose agar (PDA) culture medium. This antifungal activity was due to the fact that the CNTs caused water loss and partial or localized plasmolysis in the fungal spores. Sawangphruk et al. [40] reported the inhibition of the growth of the fungi *Aspergillus niger*, *Aspergillus oryzae,* and *Fusarium oxisporum* when adding 50, 100, and 500 μg ml^−1^ of graphene oxide in the PDA culture medium.

In crop plants, CNTs can generate ROS production after uptake and penetration into the plant, and although their high level is potentially harmful to plant cells, their production could play a role in stress perception and protection. ROS neutralizing systems include enzymatic and non-enzymatic systems that change the cell from the stressed to unstressed phase. The non-enzymatic system includes glutathione, flavonoids, carotenoids, etc. [41]. In this study, an increase in the secondary metabolites vitamin C and flavonoids was observed in tomato plants treated with CNTs and inoculated with *A. solani* compared to those that only had the pathogen. Likewise, a decrease in the severity of *A. solani* was observed in this same treatment, which may be due to the increase in these metabolites. Phenolic compounds (such as phenols and flavonoids) are characterized by having antimicrobial activity since they are related to the inhibition of the germination of fungal conidia, since they can inactivate the synthesis of essential amino acids of pathogens [42]. Ghorbanpour and Hadian [43] reported the increase in the content of flavonoids in calluses of the medicinal plant *Satureja khuzestanica* after the application of CNTs (25–1000 μg mL^−1^) to Gamboge’s B5 culture medium. However, the phenols content only increased with the application of 50, 100, and 200 μg mL^−1^ compared to the controls. Liné et al. [12] did not observe significant differences between treatments in the content of phenols in tomato, rapeseed, cucumber, and corn plants with the application of CNTs (100 mg kg^−1^ of soil). This indicates that the route of application must be considered to obtain the desired results.

Carbon nanomaterials (CNMs) have been shown to increase tolerance to various diseases, for example, the foliar application of 200 mg L^−1^ of Fullerene (C_60_) and carbon nanotubes in *Nicotiana benthamiana* plants inhibited the replication of the tobacco mosaic virus (TMV) and limited its spread to the apical tissues. In addition, the immunity of the plant was improved, the photosynthetic performance increased, as well as the activity of antioxidant enzymes and phytohormones related to defense [44]. Hao et al. [45] demonstrated that the foliar application of 200 mg L^−1^ of multi-walled carbon nanotubes (MWCNT) and reduced graphene oxide (rGO) nanoparticles inhibited the development of the pathogen *Podosphaera pannosa* that causes powdery mildew in roses (*Rosa rugosa* Thunb.).

The ROS neutralizing enzyme system includes ascorbate peroxidase, catalase, glutathione reductase, superoxide dismutase, dehydroascorbate reductase, glutathione-S-transferase, and glutathione peroxidase [41]. In this study, an increase in the activity of the GPX enzyme was observed, one of the main enzymes responsible for suppressing H_2_O_2_ to prevent cell damage in plants under various stress conditions, both biotic and abiotic. This enzyme also participates in the regulation of cellular redox homeostasis by modulating the thiol-disulfide balance [46].

The production of ROS in plants also induces the production of phytohormones, including hormones related to plant stress such as abscisic acid, salicylic acid, and jasmonic acid that provide greater defense against pathogens and herbivores to the plant. In addition, not only plants respond to immediate stress, but also make the response optimal when other stressors arrive [47]. Furthermore, CNTs can influence biochemical and physiological traits through changes in photosynthesis and a positive regulation of genes that respond to the stress and activation of plant defense systems [48].

The positive effects on the growth and development of plants after exposure to CNTs are due to the fact that they promote morphological development and the accumulation of biomass of leaves, stems, and roots through the positive regulation of genes related to the development of plants, roots, and auxin content [49]. In addition, it has been shown that CNTs can activate the expression of water channel proteins (aquaporins) that are key regulators of plant growth and development [50]. It has also been shown that CNTs improve the absorption of nutrients and water in plants due to a positive interaction between ions-plant-CNTs that retain the ions in the roots and promote the flow of water that facilitates a greater absorption of ions [9]. Once CNTs penetrate cell walls, they accumulate in plant tissues and cells and move from roots to stems and leaves, influencing cell growth and elongation due to the fact that they promote the expression of marker genes of cell division (*CycB*) and cell wall elongation (*NtLRX1*) [51].

Tripathi and Sarkar [52] showed that once present within the vascular tissue of *Cicer arietinum* plants, CNTs increase the efficiency of water and nutrient uptake which resulted in improved plant growth. Liné et al. [12] reported that under soil conditions the application of CNTs (100 mg kg^−1^ of soil) acts differently depending on the plant species. The *Brassica napus* L. and the *Cucumis sativus* L. showed an increase in biomass and the leaf area index, while the *Zea mays* L. showed symptoms of phytotoxicity since the height and fresh biomass decreased, and in the case of tomato there were no changes. Rahmani et al. [10] reported that the foliar application of CNTs (10–100 mg L^−1^) promotes the growth of *Salvia verticillata* seedlings and the production of biomass. However, concentrations of 250–1000 mg L^−1^ were toxic for this same species.

When CNTs are applied foliarly, they penetrate epidermal cells through the cuticle layer and stomatal pores [10]. From there they can reach the chloroplasts where they can increase their number and size, making the photosynthetic process more efficient [53]. Moreover, in chloroplasts they are housed within the lipid envelope where they promote the photosynthetic activity since they improve the maximum speeds of electron transport, and allow higher rates of electron transport through increased photo absorption [53]. CNMs have been shown to increase the activity of photosystem I and the enzyme ribulose bisphosphate carboxylase oxygenase (RUBISCO), which translates to more vigorous and healthy plants [54]. In addition, CNTs within the chloroplast can have other effects: They can induce the production of chlorophylls and carotenoids, and they can also act as a carbon source that facilitates carbon fixation and increases the speed of electron transport, thus inducing an improvement in photosynthesis [55].

Lahiani et al. [55] reported that the foliar application of 100 mg L^−1^ of CNT increased the efficiency of photosynthesis in *Zea maize* plants. Fan et al. [56] reported that the addition of 50 mg L^−1^ of CNT, in vitro MS culture, increased the photosynthetic rate of *A. thaliana* plants, as well as the number of lateral roots. Rahmani et al. [10] demonstrated that the foliar application of 50 mg L^−1^ of CNT improved the photosynthetic capacity of the leaves of the *Salvia verticillata* L. plant through the increase in the levels of photosynthetic pigments, in addition to stimulating the gene expression and the activity of the antioxidant enzymes. The photosynthesis rate is a parameter that is closely related to the productivity of the plants since it is determined by the fixation and the entry of CO_2_ to the plant through the stomata, and it can easily change when the plants are subjected to some type of stress. Therefore, an improvement in this physiological characteristic can translate into an increase in the productivity of the crop or in a greater capacity to produce metabolites and defense compounds that will protect the plant against some type of stress. However, the response of fruit crop and/or biomass production is dependent on multiple factors related to plant metabolism and physiology, and not only from the photosynthetic activity. Since the photosynthates produced are used for respiration, for plant growth, and for the production of defense metabolites against different types of biotic or abiotic stress [57], as is the case in this study where the crop is affected by *A. solani*. The result of this is a balance in the use of the energy available in the plant between the different routes where it will be used, ultimately observing very clear positive effects in some aspects such as tolerance to some pathogen as *A. solani*, but without such a clear effect on the growth and development of the plant as in this study.

## 5. Conclusions

The application of carbon nanotubes in tomato plants decreased the incidence and severity of *A. solani*, in addition to increasing fruit yield. In addition, the content of ascorbic acid, flavonoids, and the activity of the GPX enzyme were increased, as well as the net photosynthesis and the efficiency of the use of water with the application of CNTs.

This indicates that CNTs can be an alternative to reduce the negative impacts of pathogens such as *A. solani* in tomato crop, due to their antifungal activity, stimulation of metabolites and defense compounds, and the induction of growth and development of the crop.

## Data Availability

Not applicable.
